# Editorial: Modeling for Prediction of Radiation-Induced Toxicity to Improve Therapeutic Ratio in the Modern Radiation Therapy Era

**DOI:** 10.3389/fonc.2021.690649

**Published:** 2021-05-26

**Authors:** Tiziana Rancati, Claudio Fiorino, Giuseppe Sanguineti, Riccardo Valdagni, Ester Orlandi

**Affiliations:** ^1^ Prostate Cancer Program, Fondazione IRCCS Istituto Nazionale dei Tumori, Milan, Italy; ^2^ Unit of Medical Physics, San Raffaele Hospital, Milan, Italy; ^3^ Unit of Radiation Oncology, Regina Elena National Cancer Institute (IRCCS), Rome, Italy; ^4^ Department of Oncology and Hemato-Oncology, Università degli Studi di Milano, Milan, Italy; ^5^ Unit of Radiation Oncology 1, Fondazione IRCCS Istituto Nazionale dei Tumori, Milan, Italy; ^6^ Radiation Oncology Clinical Department, National Center for Oncological Hadrontherapy (CNAO), Pavia, Italy

**Keywords:** radiotherapy, toxicity, modeling, normal tissue complication probability, dose-response

## Introduction

Radiation therapy (RT) represents a mainstay of treatment for many cancer types, either as a single modality or within a multidisciplinary approach, including surgery and systemic therapy. From a general perspective, when planning a curative radiotherapy course, its potential benefits should be weighed against the risk of acute and late tissue/organ damage. In other words, the main goal of radiotherapy is to improve the clinical outcome by increasing the therapeutic ratio, i.e., the ratio between tumor control probability (TCP) and normal tissue complication probability (NTCP). Although modern radiotherapy techniques, such as Intensity Modulated RT (IMRT), often coupled with advanced in-room imaging (Image Guided RT, IGRT), Stereotactic Body RT (SBRT), particle RT, including proton ion and carbon ion RT, allow a better sparing of normal tissues due to their improved conformity and precision, radiation-induced toxicity is still a matter of concern. Indeed, dose tolerance of many healthy tissues, called organs at risk, is a little less than or equal to the dose needed to eradicate cancers.

It is acknowledged that the risk of some induced side effects during and after the course of curative radiotherapy may be related to radiation doses delivered to multiple organs at risk rather than to the dose received by a specific organ. Additionally, various patient-related factors, including comorbidities and genetic, genomic and biological/microenvironment features, may act as modifiers of the dose-response curve. Thus, predicting toxicity by analyzing the relationship among all determinants of radiation response of healthy tissues could improve the therapeutic ratio and the management of side effects.

The QUANTEC (Quantitative Analyses of Normal Tissue Effects in the Clinic) collaboration (1) presented a synthesis of data and models available in 2010. It derived recommendations based on what we knew at that moment. The document gave clear and exhaustive recommendations in the (few) situations where consistent results were available. In the case of controversial results or still more of a lack of reliable information, the document critically discussed the controversial points, often suggesting urgent lines of research and giving clear warnings around the uncertainty of the proposed recommendations.

During the “post-QUANTEC” years, the field’s progress has been relevant, confirming its vitality, with many research groups continuously contributing with ideas and new data. Besides, new challenges entered into the arena, substantially modifying the traditional aspects dealing with clinical dose-volume effects studies (2).

Among them, probably the most important is the shift from NTCP dose-based modelling to the broader field of more “comprehensive” predictive models. In the hypothetical case that two patients receive exactly the “same dose distribution”, the risk of toxicity is always modulated by the single individual profile.

The fact that “dose is not enough” was clear from the early days of radiobiology. It is receiving constantly growing attention in the current “omics” era (3): the availability of individual information characterizing the patients and potentially influencing their reaction to radiation is more and more essential, especially in the era of image-guided IMRT in which organs are efficiently spared in most patients.

This implies the need to have access to data including individually assessed clinical, biological and genetic information and to face the issue of modeling the response of normal tissue to radiation in a more and more “phenomenological” approach (4), requiring robust methods for the selection of the most predictive variables (both dosimetric and non-dosimetric) and the adoption of advanced data mining/machine learning methods to manage large databases, including a large number of patients and lots of variables.

Treatment planning optimization is driven by the knowledge, often not exhaustive, of quantitative dose-volume effect relationships. NTCP models are also increasingly used in protocols of model-based selection of patients for proton therapy (5–7), impacting both the single patient treatment and National Health Systems (efficiency and costs). Therefore, every progress in this field has a vast and rapid impact on how patients are treated everywhere. This is an active field of research and practice, involving many radiation oncologists, medical physicists, biologists, and data scientists in a multiprofessional scenario.

## Topics Covered in This Research Topic

This Research Topic includes Original Research Papers, Reviews, Mini Reviews and Perspective and Opinion articles focusing on:

- The state-of-the-art of modeling approaches and their contribution towards personalized cancer treatment;
- The improvements of knowledge on dose-volume relationships for different organs;
- The integration of clinical/genetic/genomic/biological/microenvironment/imaging features in prediction models;
- Pre-clinical research on radiation induced damage to normal tissues using animal models;
- Voxel-based approaches to analysis of radiation induced toxicity.

## Papers Included in This Research Topic

This Research Topic includes 30 original articles, 2 review, 1 mini-review and 1 perspective article.

The papers are from 160 authors and 18 countries on four continents. In particular, there are 19 works involving several centers and countries from one continent (10 from Europe, 6 from Asia, 2 from United States, and 1 from Australia) 9 international papers including countries both from Europe and other continents, and 6 papers from Italian centers. Authors’ affiliations are equally distributed among academies and hospitals. These summary statistics mirror the broad interest in modeling radiation-induced toxicity, the highly multidisciplinary background of people involved in the field, and the vital relationship between academic and clinical research teams.

Four pre-clinical studies are presented: McKelvey et al. consider the interaction between immunotherapy and radiotherapy, Wang et al. studied the mitigation of side-effects by removing senescent cells, Li et al. present results in mice on aerosolized thyroid hormone in preventing lung fibrosis, and Zuppone et al. propose a review of pre-clinical research on bladder toxicity

Four manuscript focus on general/methodological issues: Barry et al. evaluate the propagation of uncertainties in biologically driven treatment planning systems, Thor et al. reinforce the value of registering study analysis plans and proposes some guidelines, Isaksoon et al. review machine learning methods applied to modeling of radiotherapy outcomes, while Desideri et al. propose a mini-review on available models including radiomics features in models.

Most papers (26/34) report original research on modeling toxicity outcomes in clinical cohorts. Cancer sites include brain tumors, head-and-neck and thoracic diseases (mainly breast cancer, lung and esophageal cancers), prostate cancer. Twenty-one out 26 papers focus on photon external beam radiotherapy. At the same time, one considers proton-therapy (Palma et al.), one carbon ions (Dale et al.), one brachytherapy (Panettieri et al.) and one radioligand therapy (Belli et al.). A last work considers modeling secondary malignancy in the frame of comparison of photons and protons radiotherapy (Konig et al.). This uneven distribution is associated with a more mature experience in toxicity modeling after external beam RT; simultaneously, it highlights recent interest from the environment of more modern therapies.

Thirteen out of 26 papers consider more established modeling methods, including clinical and dosimetric risk factors (Jasper et al.; Zhao et al.; Lee et al.; Dupic et al.; Scoccianti et al.; Palma et al.; Panettieri et al.; Bresolin et al.; Onjukka et al.; Dale et al.; Belli et al.; Rattay et al.; Meng et al.). Some papers consider the inclusion of radiomics (Avanzo et al.; Du et al.), genetic information (Palumbo et al.; Massi et al.) and patient-specific biomarkers (Luo et al.; von Reibnitz et al.; Dulong et al.).

Evaluation of models including advanced dosimetric features beyond the dose-volume-histograms is presented in two papers: Heemsbergen et al. considering rectum dose maps and Marcello et al. conducting three-dimensional voxel-based analysis.

Interestingly four papers consider external validation of previously published models and or clinical/dosimetric/genetic features (Shi et al.; Panettieri et al.; Massi et al.; Rattay et al.), investigating when models can be generalized to populations other than the ones used for their training, how well this works and which cautious should be considered.

Two papers put the use of models in the perspective of modern radiotherapy: Bijman et al. consider automated radiotherapy planning to explore at the single-patient level the trade-off between tumor coverage and predicted toxicity; Lafond et al. investigate the feasibility and the added value of planning which considers specific organ sub-regions while preserving the dose to the target for prostate radiotherapy.

## Conclusions

The QUANTEC papers were published as a special issue of the Red Journal in March 2010 and became hugely successful with copies of QUANTEC dose constraints tables hanging in most dose-planning office spaces and hundreds to a thousand citations each of the published papers. However, as we passed the tenth anniversary of QUANTEC, there is a need for a renewed coordinated effort to take the use of mathematical bioeffect models for decision support and treatment plan comparison in radiation oncology to the next level for a range of reasons, including: (i) understanding that patient related risk factors may substantially impact organ tolerance, (ii) documented problems with external validation of dose-response models, (iii) more complicated associations of dose distribution to toxicity than a single dose-volume metric in a well-defined tissue structure, (iv) normal tissue effect models are being proposed for comparing competing high-cost treatment options (e.g. hadrons *vs.* photons).

The 34 papers published in this Research Topic constitute a vital contribution to the field. New interesting results are included, new topics and challenges are approached. The Research Topic witnesses the broad involvement of multidisciplinary teams towards a better understanding of the complex relationships between dose and biological response of healthy tissues, with the final aim of reaching improved optimization and personalization of radiotherapy treatments.

## Author Contributions

All authors listed have made a substantial, direct and intellectual contribution to the work, and approved it for publication.

## Conflict of Interest

The authors declare that the research was conducted in the absence of any commercial or financial relationships that could be construed as a potential conflict of interest.
